# Cancer-specific glycosylation of CD13 impacts its detection and activity in preclinical cancer tissues

**DOI:** 10.1016/j.isci.2023.108219

**Published:** 2023-10-16

**Authors:** Francis M. Barnieh, Sebastian P. Galuska, Paul M. Loadman, Simon Ward, Robert A. Falconer, Sherif F. El-Khamisy

**Affiliations:** 1 Institute of Cancer Therapeutics, Faculty of Life Sciences, University of Bradford, Bradford BD7 1DP, UK; 2 Institute for Reproductive Biology, Research Institute for Farm Animal Biology (FBN), Wilhelm-Stahl-Allee 2, Dummerstorf, Germany; 3 Incanthera plc, Manchester M2 4NH, UK

**Keywords:** Glycobiology, Molecular biology, Cancer

## Abstract

Harnessing the differences between cancer and non-cancer tissues presents new opportunities for selective targeting by anti-cancer drugs. CD13, a heavily glycosylated protein, is one example with significant unmet clinical potential in cancer drug discovery. Despite its high expression and activity in cancers, CD13 is also expressed in many normal tissues. Here, we report differential tissue glycosylation of CD13 across tissues and demonstrate for the first time that the nature and pattern of glycosylation of CD13 in preclinical cancer tissues are distinct compared to normal tissues. We identify cancer-specific O-glycosylation of CD13, which selectively blocks its detection in cancer models but not in normal tissues. In addition, the metabolism activity of cancer-expressed CD13 was observed to be critically dependent on its unique glycosylation. Thus, our data demonstrate the existence of discrete cancer-specific CD13 glycoforms and propose cancer-specific CD13 glycoforms as a clinically useful target for effective cancer-targeted therapy.

## Highlights

• Removal of CD13 glycans selectively impacts its detection and activity in cancer
• Data indicate the existence of discrete cancer-specific CD13 glycoforms
• Targeting cancer-specific proteins is an effective and safer anti-cancer approach
• Cancer-specific CD13 glycoform is a promising target for cancer-targeted therapy

## Graphical abstract

**Figure:**
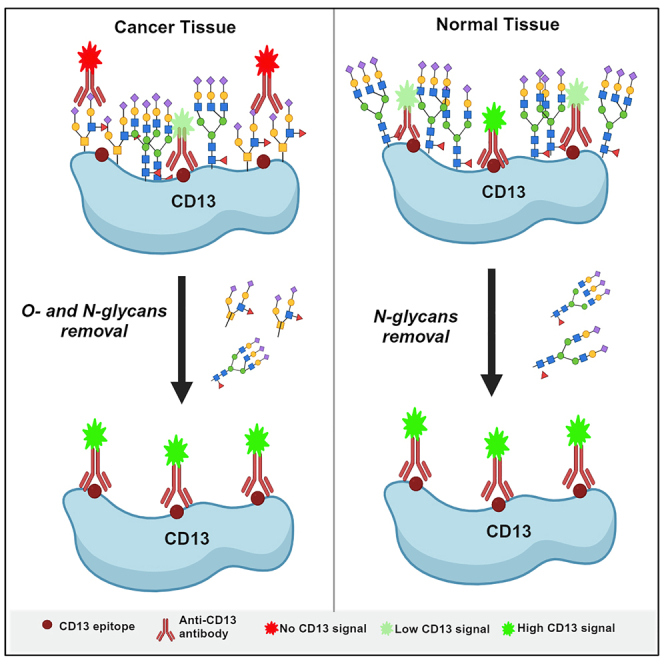

## Introduction

Given the severe side effects and toxicities often associated with current conventional cancer treatment, cancer-targeted therapies, including prodrugs and antibody conjugates of cytotoxic agents, have emerged as a primary focus in modern anti-cancer drug development.^1^^,^^2^^,^^3^ Consequently, a significant effort has been focused on identifying novel targets that deliver drugs effectively with high specificity to cancer cells, while being less toxic to normal cells. One of such targets is CD13,^4^^,^^5^^,^^6^ though it has thus far failed to fulfill its clinical potential due to the current lack of knowledge of its tissue-specific expression.^7^^,^^8^

CD13 (Aminopeptidase N, APN) is a heavily glycosylated protein, whose expression and multiplicity of function have strongly been implicated in the hallmarks of cancer, particularly tumor angiogenesis and metastasis.^8^^,^^9^ In recent years, CD13 has attracted considerable and growing interest as a target for novel anti-cancer drug development, considering its now-established critical roles in cancer progression.^4^^,^^10^^,^^11^^,^^12^ However, despite the well-reported evidence of CD13 over-expression and activity in cancer progression, the ubiquitous nature of its expression in normal tissues as generally reported remains a limitation to the full potential clinical utility of CD13 as a target for cancer therapeutics and drug discovery being exploited.^9^

CD13 is regarded as a ubiquitously expressed glycoprotein, yet its multiple functions (often termed “moonlighting activities”), as a peptidase, receptor, or signaling molecule, are well established as tissue dependent.^5^^,^^8^^,^^13^^,^^14^ It remains poorly understood as to how the same protein can function differently in cancers compared to normal tissues. The concept of the possible existence of tissue-specific distinct CD13 isoforms or glycoforms present in different tissues has long been postulated, though this remains unproven.^5^^,^^15^^,^^16^^,^^17^

Glycosylation is a common posttranslational modification of proteins and is known to contribute significantly to the generation of different forms of a protein with altered localization and function.^18^^,^^19^ The glycosylation of CD13 accounts for about 20%–40% of its observed molecular weight (240–150 kDa), with several distinct glycoforms of CD13 that differ in size reported to be present in a single cell due to variations in glycosylation.^14^^,^^15^ Unfortunately, the implications of this differential glycosylation on CD13 expression and function, particularly in a tissue-specific manner, remain unestablished. Altered protein glycosylation is a common feature of cancer cells, which leads to modulation of structure and conformation, structure and functional activity of cancer-associated glycoprotein as compared to their normal healthy tissue counterparts.^20^^,^^21^ We therefore hypothesized that the glycosylation of CD13 is tissue specific, with cancer tissues-expressed CD13 differentially glycosylated as compared to normal tissues. The identification of cancer-specific glycoforms presents a novel targeted therapeutic opportunity for the development of cancer-targeted therapeutics, which improve the healthcare outcomes and quality of life for cancer patients and their families.

## Results

### Validation of available CD13 antibodies with different epitope-binding CD13

**Figure 1:**
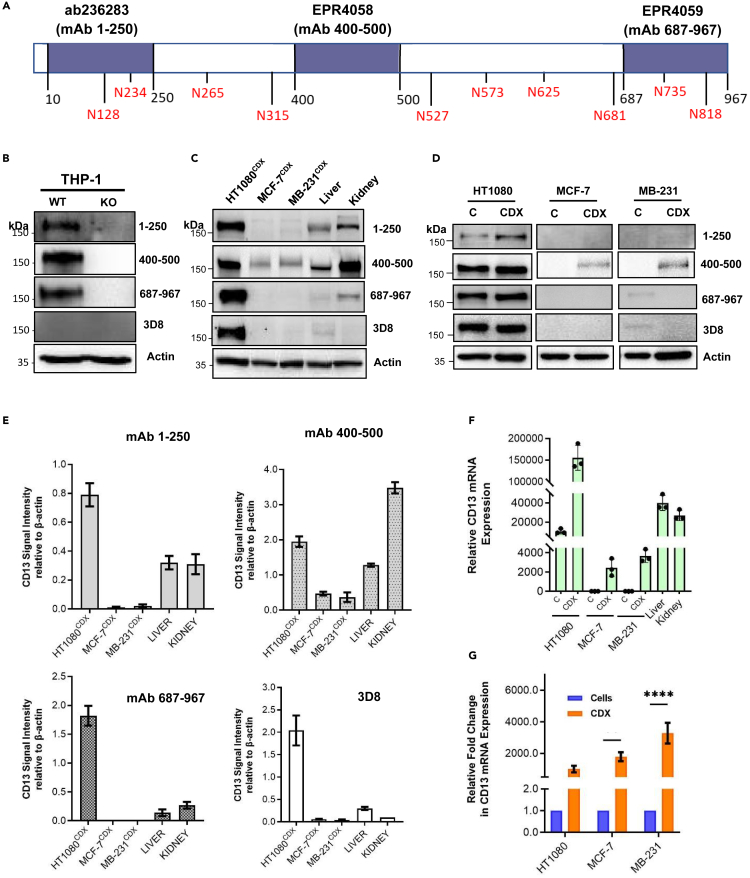
Expression of CD13 in cancer cell, CDX, and normal tissues (A) Schematic diagram of CD13 structure showing N-glycosylation sites and epitopes on the protein where the anti-CD13 antibodies used bind. (B) Reactivity of anti-CD13 antibodies to CD13 validated in CD13-KO THP-1 cells. CD13 expression in human cancer CDX and normal tissues (liver and kidney) using different epitope-binding CD13 antibodies; (C) Western blot and (E) quantified relative expression of CD13 in cancer CDX as detected by the different anti-CD13 antibodies. Band intensity measured by Image Lab Software 6.1 Software 6.1 and normalized to a β-actin loading control (D) CD13 expression in cancer cells and respective xenografts using different epitope-binding CD13 antibodies. (F) CD13 mRNA expression in cancer cells, [C], and respective xenografts, [CDX], and normal tissues. (G) CD13 mRNA expression in cancer cells, [C] and respective xenografts [CDX], and normal tissues Data shown are the mean of 3 independent experiments ±SEM. ∗∗p > 0.01 and ∗∗∗∗p > 0.0001 (two-way ANOVA).

The expression of CD13 across various tissues both in normal and cancers has been widely reported, though results are contradictory and highly dependent on the antibody and detection technique used. Hence, we first validated the reactivity of the antibodies used in this study to CD13 expression. Three commercially available CD13 antibodies, which recognize specific epitopes within the N-terminal [mAb 1–250], mid-region [mAb 400–500], and C-terminal [mAb 687–967] of the CD13 protein, (Figure 1A), in addition to 3D8 were used in this study. The 3D8 epitope is unknown, though it remains a highly published anti-CD13 antibody^16^^,^^22^^,^^23^ The reactivity of the antibodies was tested using a CD13-CRISPR knockout THP-1 cell line. Anti-CD13 antibodies, mAb 1–250, mAb 400–500, and mAb 687–967, demonstrated reactivity to CD13 expression (∼160 kDa) in CD13 wild-type THP-1 cells, but not in CD13 knockout THP-1 cells. Interestingly, 3D8 showed no reactivity to CD13 expression in THP-1 wild-type cells, despite CD13 expression in these cells being detected by the other antibodies (Figure 1B), and mRNA expression being confirmed (Figure S1).

### CD13 expression in tissues varies depending on the antibody epitopes

Next the expression of CD13 in homogenized HT1080, MCF-7, and MDA-MB-231 cell line-derived xenograft (CDX) tissues, and normal mouse tissues (liver and kidney) was assessed *ex vivo* using the four anti-CD13 antibodies. We observed an inconsistent tissue expression profile of CD13 across the antibodies. All antibodies including 3D8 (which failed to detect CD13 in THP-1 wild-type cells) detected CD13 of similar size (∼160 kDa) in HT1080^CDX^. CD13 expression in liver (∼150 kDa) and kidney (∼160 kDa) tissue homogenates was detected by mAb 1–250, 400–500, and 687–987, but not 3D8. Interestingly, the expression of CD13 (∼165 kDa) in breast cancer CDX (MCF-7^CDX^ & MDA-MB-231^CDX^) was faintly detected only by mAb 400–500 (Figures 1C and 1E). However, contrary to the observed antibody-dependent CD13 detections in cancer CDX tissues and normal tissues at the protein level, CD13 mRNA was detected in all examined tissues (Figure 1F).

We also evaluated the expression of CD13 in cancer cells and compared this to the corresponding xenograft tissue. Consistent with the lack of CD13 mRNA expression in MCF-7 and MDA-MB-231 cells (Figure 1F), none of the examined antibodies significantly detected CD13 protein in these cells (Figure 1D), although it is worth stating that mAb 687–967 and 3D8 detected very faint CD13 expression in MDA-MB-231 cells but not in MDA-MB-231^CDX^. A significantly high fold increase both at mRNA level, >2000-fold (Figure 1G), and protein level, >60-fold, (detected by only mAb 400–500, Figure S2), was observed with MCF-7^CDX^ and MDA-MB-231^CDX^ homogenate tissues compared to their respective cancer cells. With the exception of mAb 1–250, which demonstrated about 2-fold relative CD13 protein expression in HT1080^CDX^ compared to the detection of CD13 in HT1080 cells, no significant change in CD13 protein expression was observed in HT1080 cells and respective CDX by mAb 400–500, mAb 687–967, and 3D8 (Figure S2), despite an approx. 1,000-fold increase in mRNA expression. (Figure 1G).

### Glycosylation of CD13 masks detection of tissue-specific glycoforms

The observed inconsistent CD13 detection by the different epitope-binding antibodies in the examined tissues is indicative of tissue-specific posttranslational modifications that generate tissue-specific forms of CD13 with differential reactivity to the antibodies. To test this hypothesis, we investigated the direct influence of glycosylation on the reactivity of the antibodies. First, we examined the effect of N-glycosylation on the reactivity of the antibodies. Peptide:N-glycosidase F (PNGase-F) is an endo-glycosidic enzyme that efficiently removes all N-glycans from a glycoprotein.^24^ Tissue samples were treated with PNGase-F before analysis by western blot.

**Figure 2:**
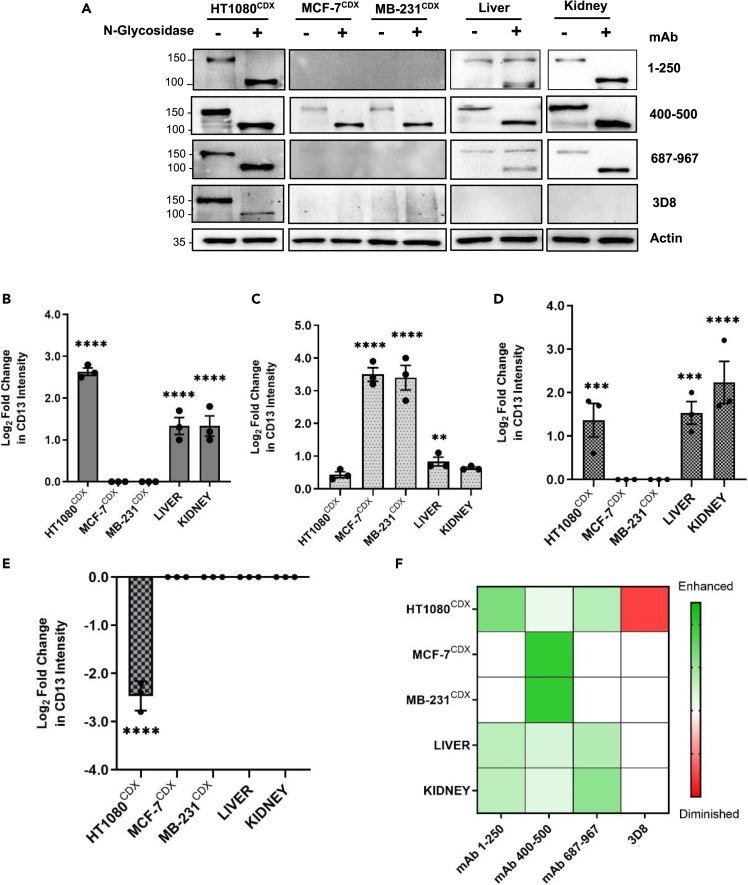
Differential effect of N-de-glycosylation on CD13 antibody reactivity in human cancer CDX and normal tissues (A) Western blot analyses and (B–E) quantified fold change of the reactivity of anti-CD13 antibodies on CD13 expression in human cancer CDX and normal tissue after N-de-glycosylation in mAb 1–250 (B), mAb 400-50 (C), mAb 687–967 (D), and 3D8 (E), respectively. Mean fold change in protein band intensity was measured using Image Lab Software 6.1 and normalized to a β-actin loading control (F). HeatMap of anti-CD13 antibody reactivity to CD13 in human cancer CDX and normal tissues after N-de-glycosylation. Data shown are the mean of 3 independent experiments ±SEM. ∗∗p > 0.01, ∗∗∗p > 0.001 and ∗∗∗∗p > 0.0001 (two-way ANOVA).

Treatment with N-glycosidase significantly shifted CD13 band from ∼150–160 kDa to ∼115–120 kDa in all tissues with respective detecting antibodies, demonstrating the heavy N-glycosylated nature of CD13 (Figure 2A). Interestingly, the reactivity of the antibodies was observed to be significantly altered in a tissue-dependent manner after the removal of N-glycans. Enhanced reactivity of mAb 1–250 and mAb 687–967 to CD13 was observed only in N-glycosidase-treated HT1080^CDX^ and kidney tissues (Figures 2A, 2B, and 2D). Reactivity of mAb 400–500 was significantly enhanced in only N-glycosidase-treated MCF-7^CDX^ and MDA-MB-231^CDX^ (Figures 2A and 2C). In contrast, the reactivity of 3D8 to CD13 was observed to be significantly diminished in N-glycosidase-treated HT1080^CDX^ (Figures 2A and 2E). Meanwhile, the reactivity of antibodies in liver tissue was observed to be seemingly unaffected by PNGase-F treatment, although CD13 detection by mAb 1–250 and mAb 687–967 revealed a subset of CD13 glycoforms apparently present only in liver, which remain unaffected by N-glycosidase activity (Figure 2A). The observed differential reactivity of the antibodies to the same protein in different tissues after the removal of N-glycans (Figure 2F) is indicative of tissue-differential N-glycosylation of CD13 around the antibody recognition epitopes.

**Figure 3:**
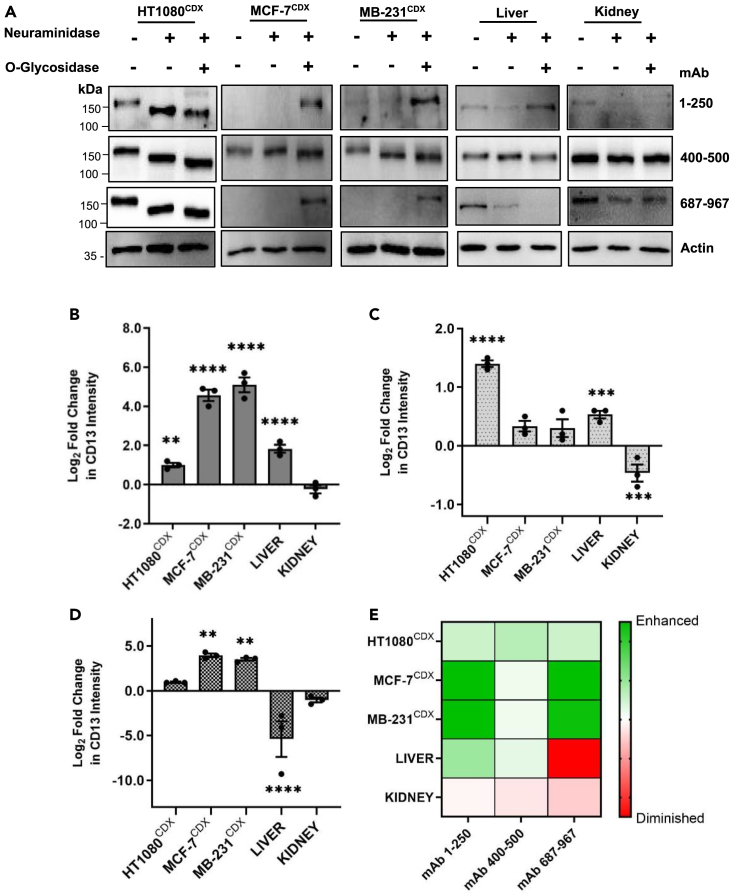
Differential effect of O-deglycosylation on CD13 antibody reactivity in human cancer CDX and normal tissues (A) Western blot analyses and (B–D) quantified fold change of the reactivity of anti-CD13 antibodies on CD13 in human cancer CDX and normal tissue after O-deglycosylation in mAb 1–250 (B), mAb 400-50 (C), and mAb 687–967 (D), respectively. Mean fold change in protein band intensity was measured using Image Lab Software 6.1 and normalized to a β-actin loading control (E). HeatMap of anti-CD13 antibody reactivity to CD13 in human cancer CDX and normal tissues after O-glycosylation. Data shown are the mean of 3 independent experiments ±SEM. ∗∗p > 0.01, ∗∗∗p > 0.001 and ∗∗∗∗p > 0.0001 (two-way ANOVA).

We next evaluated the effect of O-glycosylation on the reactivity of the antibodies. O-glycosidase releases the Core 1 and Core 3 disaccharides (Galβ1,3GalNAc and GlcNAcβ1,3GalNAc) from O-glycans, which are linked to serine or threonine residues.^25^^,^^26^ However, the presence a terminal sialic acid residue is known to hinder O-glycosidase from functioning. Hence, for efficient hydrolysis of O-glycans, a combination of neuraminidase and O-glycosidase is required.^26^ The removal of O-glycans greatly enhanced the reactivity of anti-CD13 antibodies, particularly N- and C-terminal epitope-binding antibodies mAb 1–250 (∼30- to 50-fold) and mAb 687–967 (∼12- to 18-fold), to CD13 in MCF-7^CDX^ and MDA-MB-231^CDX^ tissues despite their inability to detect the glycosylated form of CD13 (Figures 3A, 3B, and 3D).

However, in normal tissues the removal of O-glycans either did not affect the reactivity of anti-CD13 antibodies or diminished reactivity (Figures 3A–3D). The removal of O-glycans was observed to selectively enhance the detection of CD13 in breast cancer CDX tissue by the anti-CD13 antibodies, particularly those with epitope-binding domains at the terminal ends of the CD13 protein (i.e., mAb 1–250, mAb 687–697, Figure 3E). Together, these data suggest tissue-specific glycosylation profiles that pose differential structural hindrance to CD13 antibody binding to CD13 in various tissues. These findings explain the contradictory and inconsistent reported tissue expression of CD13 in the literature. This suggests CD13 is likely to exist as different glycoforms in a tissue-specific manner.

### Glycoforms of CD13 are tissue specific

**Figure 4:**
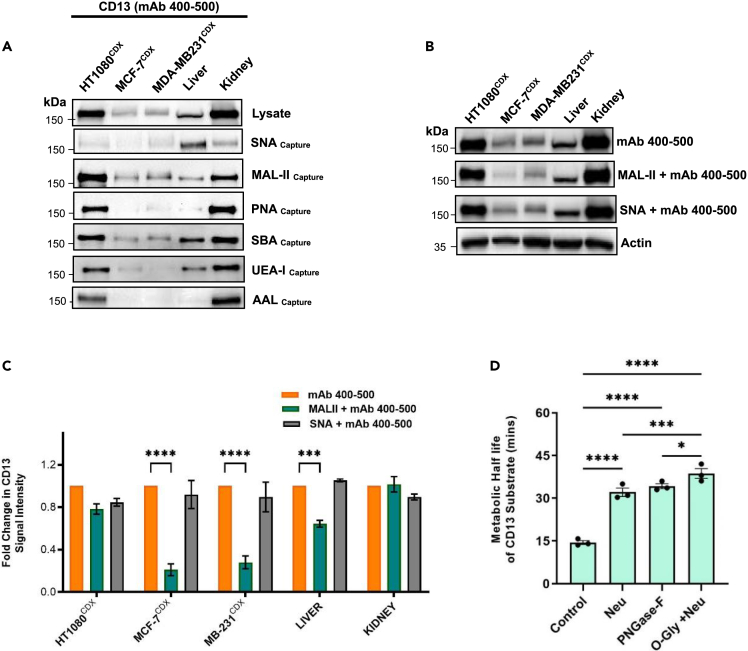
Human cancer CDX and normal tissue lysates were subjected to lectin affinity capture (A) Captured proteins were assessed by immunoblotting for CD13 using mAb 400–500 anti-CD13 antibody. Loading was assessed by Coomassie stain (Figure S4). (B and C) Effect of sialo-glycan lectin binding on CD13 detection using mAb 400–500 anti-CD13 antibody and in cancer CDX and normal tissues. (D) Significance of glycosylation on the metabolic half live (min) of CD13 substrate in MCF-7 CDX homogenate. Data shown are the mean of 3 independent experiments ±SEM. ∗∗∗p > 0.001 and ∗∗∗∗p > 0.0001 (two-way ANOVA).

To confirm the possibility that CD13 may exist as a distinct glycoforms in cancers and normal tissues, specific glycan-binding lectins (Table 1) were used to capture CD13 in cancer CDX and normal tissues. Captured CD13 glycoforms were assessed by CD13 immunoblotting using anti-CD13 antibody mAb 400–500. Although few significant differences were observed between HT1080^CDX^ and kidney tissue lectin-captured CD13 populations, populations captured in breast cancer CDX tissue (MCF-7^CDX^ & MDA-MB-231^CDX^) were significantly distinct compared to normal tissues and HT1080^CDX^ suggesting tissue-specific heterogeneous populations (glycoforms) of CD13. Focusing on the captured sialoglycans [α2,3- (MAL-II) and α2,6-linked sialic acid (SNA)] populations, CD13 present in breast cancer CDX tissue was observed to present different sialylation, particularly α2,3 sialoglycans, compared to normal tissues. In breast cancer CDX tissue, CD13 was less detected in captured α2,6-sialoglycan population though relatively prominently detected in captured α2,6-sialoglycan population. This was contrary to observations in normal tissues (Figure 4A; Table 2). This is a further confirmation of the tissue-specific manner of CD13 glycosylation, but more importantly an indication of a distinctive sialylation pattern on CD13 in breast cancer CDX tissue tissues compared to normal healthy liver and kidney. This observation appears to correlate with an observed lack of expression of α2,3 sialidases (Neu-2 and Neu-4) in breast cancer CDX tissue (Figure S3A).

**Table 1:** Lectins and their glycan-binding specificities

Lectin	Species/Origin	Nominal binding specificity
SNA	Sambucus nigra	α2,6-linked sialic acid
MAL-II	Maackia amurensis-II	α2,3-linked sialic acid
PNA	Arachis hyogaea	Galβ1,3GalNAcα-Thr/Ser
SBA	Soybean Agglutinin	βGalNAc, Gal
UEA-I	Ulex europaaeus-I	Fucα1,2GlcNAc
AAL	Aleuria aurantia	Fucα1,6GlcNAc

**Table 2:** CD13 detection in lectin-affinity captured proteins from human cancer CDX and normal tissues

	mAb 400-500					
	*SNA*	*MAL II*	*PNA*	*SBA*	*UEA*	*AAL*
HT1080^CDX^	+	+++	+++	+++	++	++
MCF-7^CDX^	+	++	–	+	+	–
MDA-MB-231^CDX^	+	++	+	+	–	–
LIVER	++	+	+	++	+	–
KIDNEY	+	++	+++	+++	++	+++

High (+++); Medium (++); Lower (+); Negative (−).

As demonstrated in Figures 2 and 3, the glycosylation of CD13 around the antibody epitopes leads to diminished recognition, likely to be due to structural hindrance to antibody binding. We therefore investigated the position of α2,3- and α2,3-sialoglycans in relation the recognition epitope of anti-CD13 antibody (mAb 400–500). This was done by incubating the immunoblot of cancer CDX and normal tissues with SNA and MAL-II lectins before the detection of CD13 using mAb 400–500. The lectin binding to sialic acids on CD13 will hinder CD13 detection if it occurs close to the mAb antibody recognition epitopes. Lectin blotting was performed to confirm the glycan affinity of lectin used (Figure S3B). In the presence of MAL-II (α2,3-linked sialic acid) lectin binding, CD13 detection by anti-CD13 antibody (mAb 400–500) was significantly reduced in breast cancer CDX tissues compared to normal tissues. However, the SNA (α2,6-linked sialic acid) lectin binding was observed to have no effect on the reactivity of mAb 400–500 to CD13 in all tissues. (Figures 4B and 4C).

## Discussion

We have demonstrated that although CD13 is heavily glycosylated in both cancers (CDX) and normal tissues (liver and kidney), the nature (identity and position) of this glycosylation differs across tissues and as such poses tissue-specific structural hindrance to the detection by CD13 antibodies depending on the epitope-binding recognition of the antibody in question. This explains the largely inaccurate and contradictory reports on the expression of CD13 in these tissues, using different monoclonal antibodies and detection techniques.^16^^,^^22^^,^^27^

Deglycosylation of CD13 before antibody detection may provide a more accurate CD13 expression profile across tissues, particularly, when using CD13 antibodies with binding epitopes within the N and C termini of the CD13 protein. The inactivation of peptidic epitopes by glycosylation as observed here has recently been reported in detection of various glycoproteins in cancer, not least in the detection of cancer immunotherapy target PD-L1.^28^^,^^29^ These data highlight the profound impact of differential tissue glycosylation of CD13 on its detection by antibodies, particularly in cancers.

We have additionally demonstrated that the frequently utilized anti-CD13 antibody that has appeared in a large number of publications,^22^^,^^30^ 3D8, whose epitope-binding characteristics remain unknown, may likely require specific glycosylation on CD13 for its reactivity (Figure 2). Thus, considering the demonstrated tissue-differential glycosylation of CD13, this may explain why the reactivity of this antibody was observed only in HT1080 CDX tissues: perhaps the required glycosylation is absent in the other examined tissues. This is not a unique phenomenon to this CD13 antibody, as similar observations have been reported with other antibodies.^29^^,^^31^ Caution should be exercised when assessing reports of CD13 expression with this antibody, particularly when comparing CD13 expression across different tissues and conditions.

CD13 is an established regulator of tumor angiogenesis and is markedly induced in hypoxia and in xenograft tumor growth; CD13 expression is induced in cancer cells that do not endogenously express the protein under these conditions.^32^ This was the case in breast cancer cells and respective xenograft tissues (Figures 1A and 1E). Importantly, however, our data demonstrate that the glycosylation of CD13 induced and expressed under tumor microenvironmental conditions, particularly in breast cancer CDX tissue, is very distinct from the glycosylation observed in endogenously CD13-expressing tissues, such as HT1080 CDX and normal tissues (liver and kidney). This observation complements a key study by Curnis et al. which demonstrated the selective binding of an asparagine-glycine-arginine (NGR) peptide motif to CD13 expressed on tumor angiogenic vessels; it did not bind to CD13 expressed in normal tissues, including kidney.^5^ The structural basis for this selective ligand binding remains unknown. However, to explain the observation, the authors ruled out possible cancer-specific glycosylation, suggesting the possibility of a CD13 isoform with a unique conformation expressed exclusively on tumor angiogenic vessels.

On the contrary, we suggest cancer-selective O- and N-glycosylation at terminals and mid regions respectively of the CD13 protein in breast cancers. This glycosylation was demonstrated to selectively block antibody reactivity to CD13 in breast cancer in a way not observed in liver and kidney. In addition, the metabolism of a tumor-specific CD13 substrate was observed to be critically dependent on the unique CD13 glycosylation in tumors (Figures 4D and S5). This finding, along with the evidence for the distinct structural differences discussed, is the first evidence to indicate that the differential tissue-specific glycosylation of CD13 is important not only for its detection but also its activity. These data also perhaps explain the many reported tissue-specific functions of CD13 despite its ubiquitous expression.^8^^,^^33^^,^^34^ Glycosylation of proteins, particularly membrane proteins, is known to be critical for ligand-receptor interactions, stability, and activities of these proteins.^35^^,^^36^ Also, the terminal epitopes of glycoproteins have been shown to play a significant role in cell-cell interactions, cell-cell and cell-matrix adhesion, and to influence cancer metastasis.^37^^,^^38^ Thus, the differential glycosylation of CD13, particularly at both the N and C termini in examined tissues as demonstrated in this study may imply specific ligands and substrates with affinity for CD13 in various tissues. This is likely to account for its tissue-specific functions.

Hyper and distinctive sialylation is known feature of cancer progression and is considered a potential therapeutic target in its own right for cancers, including breast cancer.^39^^,^^40^^,^^41^^,^^42^ Using lectin capture, we have demonstrated CD13 as a sialo-glycoprotein with differential α2,3-sialylation in cancers. Although several studies have reported differential α2,3-sialylation in metastatic breast cancer,^40^^,^^43^^,^^44^ few glycoproteins including CD44 and mucins have been identified to be responsible for this observation.^40^ Our data suggest that cancer-specific sialylation of CD13 may significantly contribute to the hyper-sialylation observed in cancers.

The distinct aberrant cancer-associated glycans on CD13 as observed may represent an important cancer biomarker and target for anti-cancer-specific therapy. Glycoform-specific targeting of proteins in recent years has been demonstrated as an effective approach to limit off-target toxicities associated with current anti-cancer therapy, while increasing the antitumor specificity.^45^^,^^46^ Thus, the application of cancer-targeting strategies including antibody-drug conjugates (ADCs),^47^ and peptide-drug conjugate (PDC),^48^ which are selective to cancer-specific glycoforms of CD13, hold significant promise for cancer-specific therapy.

In conclusion, CD13 is a target with significant unfulfilled clinical potential in cancer drug discovery due its ubiquitous expression in normal tissues.^8^^,^^9^ This study has demonstrated for the first time that tumor microenvironment-induced and expressed CD13 in breast cancers CDX carry distinct cancer-associated glycans compared to normal tissues. In addition, evidence has been provided that these differences relate to the metabolism activity of the protein. Thus, our data suggest the existence of cancer-specific glycoforms of CD13, which are selectively present in cancers, but absent in normal tissues. This work therefore provides an important foundation for further investigations to identify and fully characterize the distinctive nature of cancer-associated glycans on CD13 particularly in clinical cancer tissues, which could lead to enhanced molecular tools, diagnostic biomarkers, as well as clinically useful targeted therapeutics. Targeting cancer-specific glycoforms as demonstrated in recent times with highly specific antibodies of cancer-specific LYPD3,^46^ MUC1,^49^ and CD43^50^ glycoforms is a promising therapeutic strategy to selective cancer cells but not normal tissues. Hence, the characterization of the distinctive glycostructure and substrate specificity of cancer-specific CD13 glycoforms could enable the development of highly specific CD13 antibody and peptide substrate for potent ADCs and PDCs that target only cancer-expressed CD13 glycoforms.^47^^,^^48^ These tools hold significant potential to a better understanding of cancer-specific functions of CD13 and, ultimately, its clinical usefulness as a target for effective precision targeted therapy.

### Limitations of the study

In this present study, we have demonstrated that the glycosylation of cancer-expressed CD13 is cancer specific and distinct compared to that expressed in normal tissues. However, the clinical significance of this finding is not clear as the study was conducted using human cell lines and CDXs. Further studies in clinical cancer tissues are required to assess the clinical and therapeutic implications of cancer-specific CD13 glycosylation patterns.

## STAR★Methods

### Key resources table

**Table:** 

REAGENT or RESOURCE	SOURCE	IDENTIFIER
**Antibodies**		

CD13 Antibody (3D8)	Santa Cruz	Cat# sc-13536; RRID: AB_626894
CD13 antibody [EPR4058]	Abcam	Cat# ab108310. RRID: AB_10866195
CD13 antibody [EPR4059]	Abcam	Cat# ab108382. RRID: AB_10890797
CD13 antibody [ab236283]	Abcam	Cat# ab236283
Streptavidin-HRP [3999]	Cell Signaling Technology	Cat# 3999. RRID: AB_10830897
Anti-rabbit IgG, HRP-linked Antibody [7074]	Cell Signaling Technology	Cat# 7074. RRID: AB_2099233
Anti-rabbit IgG, HRP-linked Antibody [7076]	Cell Signaling Technology	Cat# 7076. RRID: AB_330924

**Biological samples**		

Cryopreserved mouse tissues	Institute of Cancer Therapeutics, University of Bradford, UK.	https://www.bradford.ac.uk/ict/
Cryopreserved human cancer CDX tissues	Institute of Cancer Therapeutics, University of Bradford, UK.	https://www.bradford.ac.uk/ict/

**Chemicals, peptides, and recombinant proteins**		

RPMI-1640	Merck-UK	Cat# R0883-500ML
Fetal Bovine Serum	Merck-UK	Cat# F4135-500ML
Phosphate buffered saline	Merck-UK	Cat# P2272-500ML
α2-3,6,8 Neuraminidase	New England Biolabs	Cat# P0720S
PNGase F	New England Biolabs	Cat# P0704S
O-Glycosidase	New England Biolabs	Cat# P0733S
High-Capacity cDNA Reverse Transcription Kit	ThermoFisher, UK	Cat# 4368814
RNeasy Micro Kit	Qiagen, UK	Cat# 74004
SYBR Green PCR Master Mix	Primer Design, UK	N/A
Maackia Amurensis Lectin II (MAL II), Biotinylated	Vector Laboratories, UK	Cat# B-1265-1
Sambucus Nigra Lectin (SNA), Biotinylated	Vector Laboratories, UK	Cat# B-1305-2
Peanut Agglutinin (PNA), Biotinylated	Vector Laboratories, UK	Cat# B-1075-5
Soybean Agglutinin (SBA), Biotinylated	Vector Laboratories, UK	Cat# B-1075-5
Aleuria Aurantia Lectin (AAL), Biotinylated	Vector Laboratories, UK	Cat# B-1015-5
Ulex Europaeus Agglutinin I (UEA I), Biotinylated	Vector Laboratories, UK	Cat# B-1395-1
Sambucus Nigra Lectin (SNA), Biotinylated	Vector Laboratories, UK	Cat# B-1065-2
Streptavidin-agarose	Vector Laboratories, UK	Cat# SA-5010-2

**Deposited data**		

Original Western Blots	This Paper, in Mendeley data	https://doi.org/10.17632/j3mrsxkj6w.1

**Experimental models: Cell lines**		

MDA-MB-231	ATCC	Cat# CRM-HTB-26
MCF-7	ATCC	Cat# CRL-3435
HT1080	ATCC	Cat# CCL-121
CD13 wildtype THP-1	Abcam	Cat# ab273759
CD13 knock-out THP-1 cells	Abcam	Cat# ab273759

**Oligonucleotides**		

Human beta-Actin qPCR Primer Pair	SinoBiological	Cat# HP100001
Human CD13 qPCR Primer Pair	SinoBiological	Cat# HP100141

### Resource availability

#### Lead contact

Further information and requests for resources and reagents should be directed to and will be fulfilled by the lead contact Francis M Barnieh (F.mprahbarnieh1@bradford.ac.uk).

#### Materials availability

• This study did not generate new unique reagents.

### Experimental model and study participant details

#### Cell culture

Human breast carcinoma (MCF-7), human breast adenocarcinoma (MDA-MB-231) and fibrosarcoma (HT1080) cell lines were obtained from American Type Culture Collection (ATCC). CD13 wildtype THP-1 and CD13 knock-out THP-1 cells from Abcam, UK. Cells were cultured in RPMI-1640 supplemented with 10% (v/v) foetal bovine serum, sodium pyruvate (1 mM), and l-glutamine (2 mM) in a humidified incubator at 37°C with 5% of carbon dioxide. Cell lines were used at low passage (<12 passages) for <6 months. Cryopreserved mouse liver, kidney and human cancer CDX were obtained from Institute Cancer Therapeutics, UK.

### Method details

#### RT-PCR

Assessment of CD13 gene expression as determined by quantitative reverse transcription-PCR. RNA was extracted from cells and tissues using RNeasy Micro Kit (Qiagen). Complementary DNAs (cDNAs) were produced using High-Capacity cDNA Reverse Transcription Kit (ThermoFisher, UK). qPCR was performed using SYBR Green PCR Master Mix (Primer Design, UK). The β-actin gene was used as an endogenous control. CD13 primer (HP100141) and β-actin primer (HP100001) were used.

#### Western and lectin blotting

*Tissue preparation****:*** Cryopreserved human cancer CDX tissues; MCF-7, MDA-MB-231 and HT-1080 CDX, and normal mouse tissues, (liver, and kidney) were homogenised in cold lysis buffer (1 in 4, w/v) with protease inhibitors. Homogenates were incubated on ice for 30 min, centrifuged (10,000 g, 10 min, 4°C) and the supernatant pipetted and stored at -80°C as the total tissue lysate.

*Cell preparation:* Cell pellets were lysed by suspending in IP lysis buffer with protease inhibitors and incubated on ice for 30 min, sonicated and centrifuged (10,000 g, 10 min, 4°C). Supernatants were pipetted and stored at −80°C as the total cell lysate. Total protein concentrations of lysate were determined using a BCA protein assay kit (ThermoFisher Scientific, UK).

Protein (40 μg) of total lysate was separated by 6-8% SDS-PAGE and then transferred onto nitrocellulose or PVDF membrane. Membranes were blocked with 5% dry milk in TBST or 3% BSA for lectin binding assay. Membranes were probed for specific protein or glycan expression with respective antibodies / biotinylated lectin, followed by horseradish peroxidase (HRP)-conjugated secondary antibodies or streptavidin-HRP for lectin blotting. These immunoblots were visualised and analysed using ChemiDoc Imaging System with Image Lab Software 6.1.

#### Lectin affinity capture

For lectin enrichment assay, 50 μl of biotinylated lectin (2 mg/ml) was added to 500 μg of tissue lysate and volume was made up to 500 μl with phosphate buffered solution (PBS). For PNA, SBA, UEA-I and AAL lectins, 1 mM MnCl_2_, 1 mM MgCl_2_, and 1mM CaCl_2_ were added. The mixture was incubated at 4°C overnight with rotation. Prewashed streptavidin-agarose (50 μl) was added and incubated was for another 4 h. The beads were washed after centrifugation and subsequently separated with 8% SDS-PAGE after boiling at 95°C for 5 min. The samples were transferred to nitrocellulose filter membranes and then the membranes were incubated with CD13/APN antibodies.

#### Deglycosylation of CD13 protein for immunoblotting

Tissue lysate samples were denatured using glycoprotein denaturing buffer (New England Biolabs). Denatured samples were then deglycosylated accordingly. For N-glycans deglycosylation, 10% NP-40, Glycobuffer 1 and PNGase-F (New England Biolabs) were added to denatured tissue lysate and incubated at 37°C for 3 h. For O-glycans deglycosylation, 10% NP-40, Glycobuffer 2, O-Glycosidase (Endo-α-N-Acetyl-galactosaminidase) and Neuraminidase (New England Biolabs) were added to denatured tissue lysate and incubated at 37°C for 4 h. Reaction mixtures were cooled and separated by 8% SDS-PAGE before Western blotting with CD13 antibodies.

### Quantification and statistical analysis

All results were expressed as mean ± standard error of measurement (SEM) from at least 3 independent experiments. The statistical analysis was performed using GraphPad Prism 8 (GraphPad Software, San Diego, CA, USA). P < 0.05 was considered statistically significant.

## Data Availability

• All data reported in this manuscript are available from the lead contact without restriction. Original western blot images have been deposited at Mendeley and are publicly available as of the date of publication. The DOI is listed in the key resources table. • No original code was generated in this paper. • Any additional information required to reanalyze the data reported in this paper is available from the lead contact upon request.
