# Supplementary material for: Cancer-specific glycosylation of CD13 impacts its detection and activity in preclinical cancer tissues
Source: iScience. 2023 Oct 16;26(11):108219. doi: 10.1016/j.isci.2023.108219 (PMC10628746; doi:10.1016/j.isci.2023.108219)
Supplement: Document S1. Figures S1–S5 and Table S1 [file mmc1.pdf]

iScience, Volume *26*

Supplemental information

Cancer-specific glycosylation of CD13 impacts its detection and activity in preclinical cancer tissues

Francis M. Barnieh, Sebastian P. Galuska, Paul M. Loadman, Simon Ward, Robert A. Falconer, and Sherif F. El-Khamisy

Supplementary Information

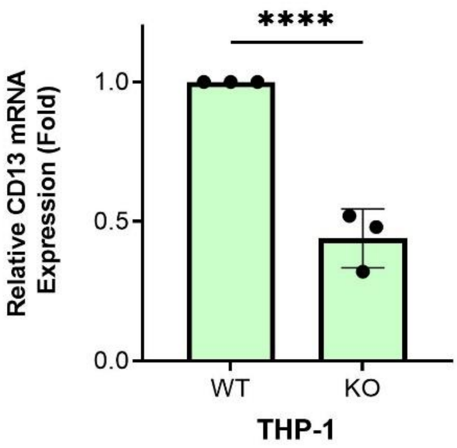

Fig. S1. CD13 mRNA expression in CD13-CRISPR knockout THP-1 cells relative to expression in THP-1 wildtype cells

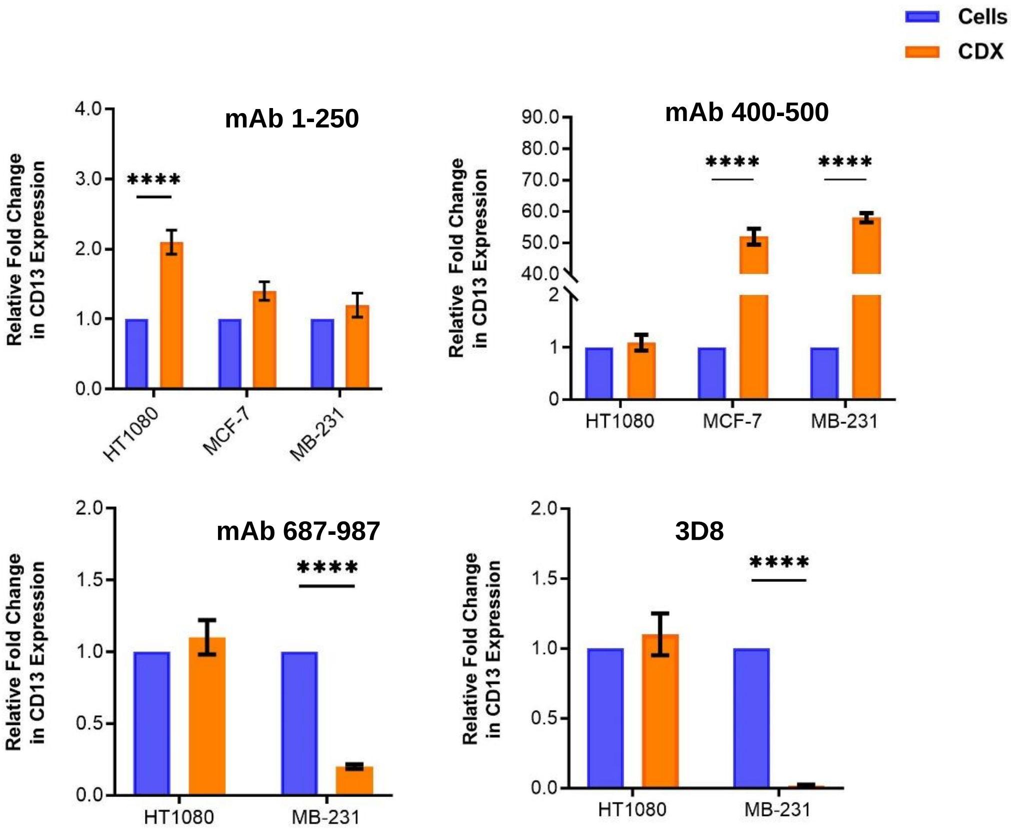

Figure S2. Quantified fold-change of CD13 protein expression in tumour xenograft relative to expression in their relative cell lines using different epitope-binding anti-CD13 antibodies. Mean fold-change in protein band intensity was measured using Image Lab Software 6.1 and normalised to a β-actin loading control.

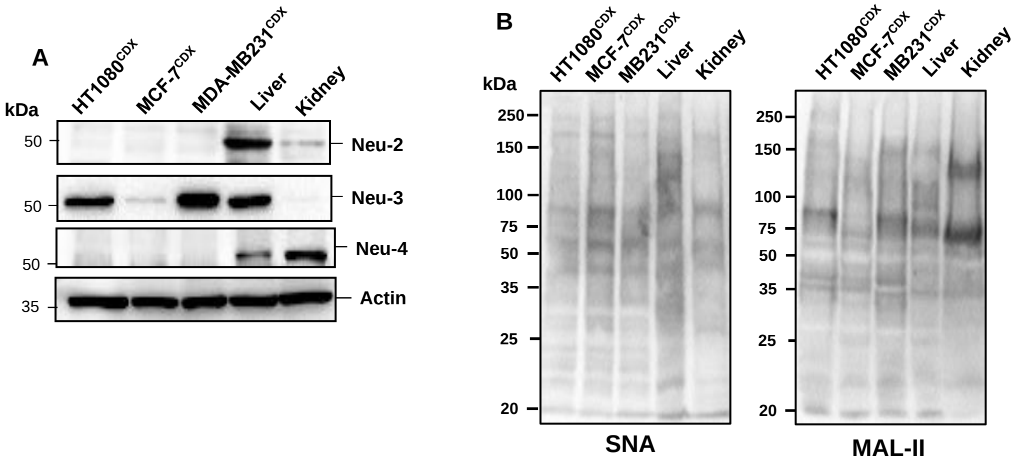

Figure S3. Expression of neuraminidases and sialo-glycans in CDX and normal tissues (A) Differential expression of neuraminidases in tumour xenografts and normal tissues. (B) SNA and MAL-II lectin blot of tumour xenograft and normal tissues, to assess their binding affinity to α2,3 and α2,6 sialo-glycans respectively.

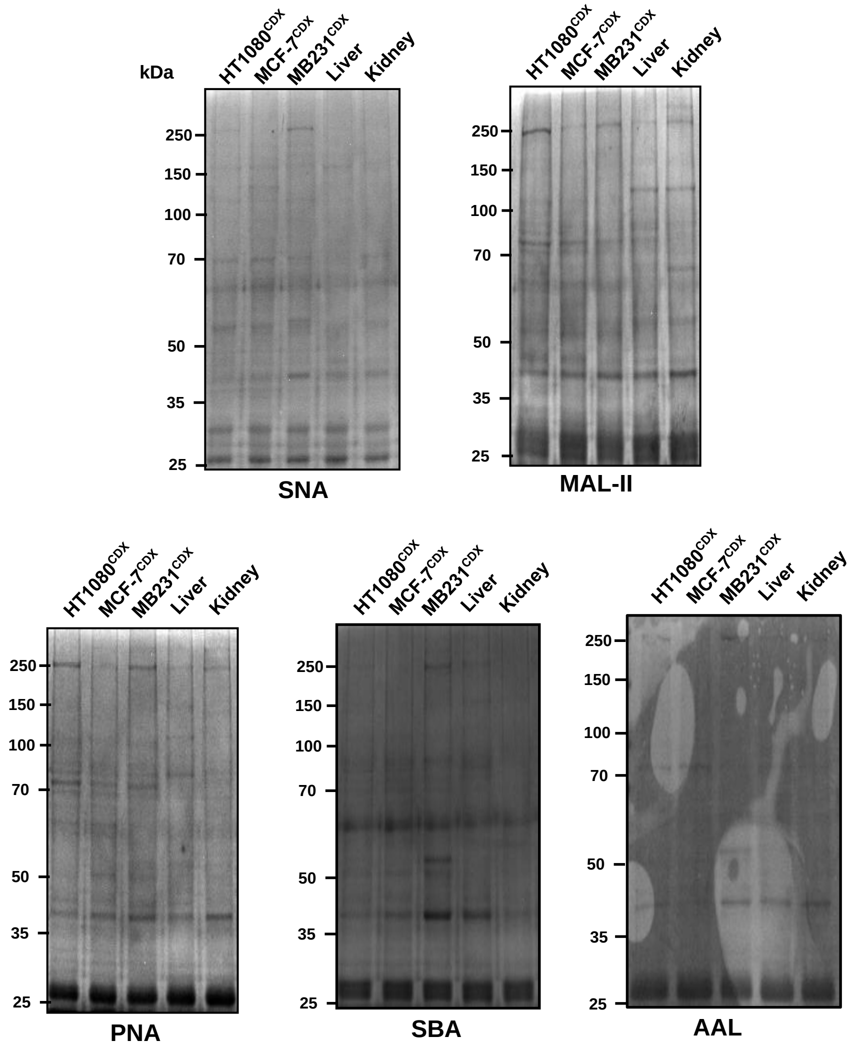

Figure S4. Coomassie staining of lectin affinity captured proteins in tumour xenograft and normal tissues.

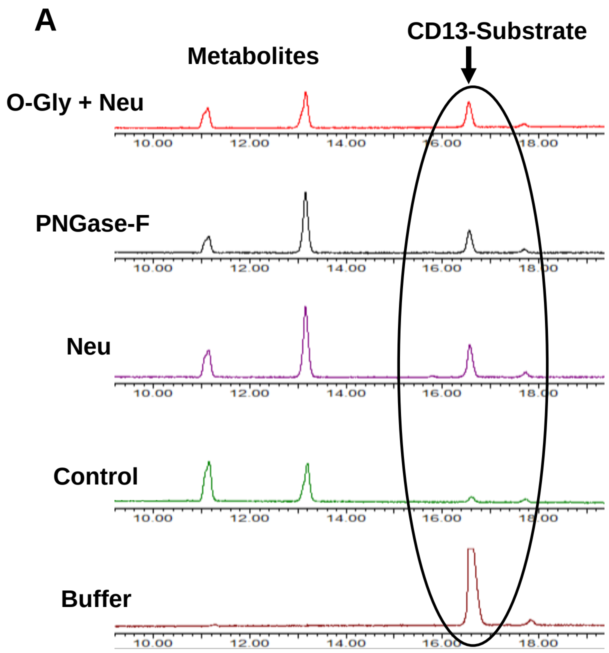

Figure S5. LC-MS spectra of CD13 substrate metabolism in MCF-7 CDX homogenate in the presence specific glycosidases.

Table S1. Lectins used in the study.

Lectin	Catalogue Number
**Maackia Amurensis Lectin II (MAL II), Biotinylated**	B-1265-1
**Sambucus Nigra Lectin (SNA), Biotinylated**	B-1305-2
**Peanut Agglutinin (PNA), Biotinylated**	B-1075-5
**Soybean Agglutinin (SBA), Biotinylated**	B-1015-5
**Aleuria Aurantia Lectin (AAL), Biotinylated**	B-1395-1
**Ulex Europaeus Agglutinin I (UEA I), Biotinylated**	B-1065-2
